# Novel functional view of the crocidolite asbestos-treated A549 human lung epithelial transcriptome reveals an intricate network of pathways with opposing functions

**DOI:** 10.1186/1471-2164-9-376

**Published:** 2008-08-07

**Authors:** Joan M Hevel, Laura C Olson-Buelow, Balasubramanian Ganesan, John R Stevens, Jared P Hardman, Ann E Aust

**Affiliations:** 1 Department of Chemistry and Biochemistry, Utah State University, Logan, USA; 2 Center for Integrated BioSystems, Utah State University, Logan, USA; 3 Department of Mathematics and Statistics, Utah State University, Logan, USA

## Abstract

**Background:**

Although exposure to asbestos is now regulated, patients continue to be diagnosed with mesothelioma, asbestosis, fibrosis and lung carcinoma because of the long latent period between exposure and clinical disease. Asbestosis is observed in approximately 200,000 patients annually and asbestos-related deaths are estimated at 4,000 annually[1]. Although advances have been made using single gene/gene product or pathway studies, the complexity of the response to asbestos and the many unanswered questions suggested the need for a systems biology approach. The objective of this study was to generate a comprehensive view of the transcriptional changes induced by crocidolite asbestos in A549 human lung epithelial cells.

**Results:**

A statistically robust, comprehensive data set documenting the crocidolite-induced changes in the A549 transcriptome was collected. A systems biology approach involving global observations from gene ontological analyses coupled with functional network analyses was used to explore the effects of crocidolite in the context of known molecular interactions. The analyses uniquely document a transcriptome with function-based networks in cell death, cancer, cell cycle, cellular growth, proliferation, and gene expression. These functional modules show signs of a complex interplay between signaling pathways consisting of both novel and previously described asbestos-related genes/gene products. These networks allowed for the identification of novel, putative crocidolite-related genes, leading to several new hypotheses regarding genes that are important for the asbestos response. The global analysis revealed a transcriptome that bears signatures of both apoptosis/cell death and cell survival/proliferation.

**Conclusion:**

Our analyses demonstrate the power of combining a statistically robust, comprehensive dataset and a functional network genomics approach to 1) identify and explore relationships between genes of known importance 2) identify novel candidate genes, and 3) observe the complex interplay between genes/gene products that function in seemingly different processes. This study represents the first function-based global approach toward understanding the response of human lung epithelial cells to the carcinogen crocidolite. Importantly, our investigation paints a much broader landscape for the crocidolite response than was previously appreciated and reveals novel paths to study. Our graphical representations of the function-based global network will be a valuable resource to model new research findings.

## Background

Asbestos is a family of naturally occurring silicate minerals that was once used extensively in a variety of building materials and industries and is still found in older structures. Exposure to certain forms of asbestos, such as crocidolite and amosite, have been shown to cause mesothelioma, asbestosis, fibrosis and carcinoma of the lungs, esophagus and stomach [2-4]. Many developing countries continue to mine and use asbestos, presenting a continued risk to individuals.

The biodurability and chemical reactivity of *crocidolite *asbestos, taken together, create a formidable carcinogen for the human lung to handle. Crocidolite can induce DNA strand breaks and base alterations. One expected response to this damage is apoptosis/cell death. But under certain conditions, cell replication can occur before the DNA damage is repaired, resulting in the formation of mutations. Events which promote survival of the cell with DNA damage and stimulate replication may lead to cancer. An unfortunate consequence of apoptosis is the stimulation of surrounding cells to replicate in an effort to repair the integrity of the damaged tissue. If the surrounding cells have experienced DNA damage, the result could be mutations, which may lead to cancer. What sets crocidolite apart from most other carcinogens is the persistent nature of the inhaled fibers, allowing for continued damage to surviving cells throughout the lifetime of the individual. Therefore, knowledge of the delicate balance between pathways that lead to proliferation or survival and those which lead to apoptosis or cell death are crucial for understanding the etiologies behind several asbestos-induced lung disorders and diseases.

Much of the deleterious effects of asbestos can be attributed to the sustained synthesis of reactive oxygen species (ROS) which in turn results in DNA damage [5-7] and oxidative stress within the cell. Iron associated with the fibers (up to 27% by weight in crocidolite) can participate in Fenton and Haber-Weiss chemistry and therefore plays an intimate role in ROS generation (reviewed by [8]). Signals which reduce glutathione synthesis and increase efflux of reduced glutathione result in the reduction of intracellular glutathione concentrations [9], thus, exacerbating the situation. At the crux of the decision to initiate apoptosis is a p53-dependent transcription response. Although the events upstream of p53 activation and the importance of p53 targets are not well characterized, the result of p53 activation is mitochondrial dysfunction leading to apoptosis [10]. Apoptosis prevents continued proliferation of the damaged cell, but factors released from the damaged cell can also affect nearby cells causing inflammation and proliferation. In mapping the signal cascades which are activated/deactivated by asbestos, both human and non-human cell lines of epithelial and/or mesothelial cells, and rodent animal models have been useful. Studies have identified MAPkinase [11-13], cytokines, Akt [11,14], PKC [15], p53 [10,16] and NF-κB [17,18] signaling pathways as important players. Chromosomal translocations, promoter silencing, point mutations, deletions, and/or familial genetic susceptibility are also likely to contribute to a cell's inability to respond appropriately to the genotoxic insult of crocidolite [19,20]. The number and complexity of the signaling components identified thus far mirrors the idea that the asbestos response is not only related to the presence of iron and the production of ROS, but also involves receptor-mediated processes. An unavoidable caveat of trying to combine all the current experimental data lies in the inherent differences that exist between species and within cell lines, primary cells, and tumor specimens (reviewed in [21]), and the involvement of neighboring tissue. The complexity of asbestos response and the multiple pathologies associated with the fiber would suggest the need for more systems biology approaches to the problem.

This study was conducted to begin to elucidate how the A549 human lung epithelial cell transcriptome is altered when cells are exposed to crocidolite asbestos at 6 μg/cm^2^. An experimental strategy was developed to ensure a statistically robust, comprehensive data set from which global observations and analyses of specific pathways could be made. This study extends a previous microarray experiment [22] performed at 2 μg/cm^2 ^where replicates were not available. Using the more than 2,500 genes that were differentially regulated by crocidolite in A549 cells, we were able to: 1) Statistically classify the data set based on gene ontologies. This analysis revealed significant representation by transcriptional corepressor and repressor activities. Additionally, a significant unique representation by DNA modification ontologies within a less significant DNA repair/response ontology was found; 2) Identify specific novel genes that may play a role in experimentally observed asbestos-induced responses; and 3) Use a knowledge-based network approach to reveal a highly integrated series of networks related to cell death, cancer, cell cycle, cellular growth, proliferation, and gene expression. Network analysis identified several functional modules in which previously unidentified genes may play a central role in the response of cells to asbestos, including participation from an extensive extracellular set of growth factors and cytokines. Importantly, by combining genome-wide transcript changes and functional network analysis, we have documented a novel global view of the crocidolite-treated A549 transcriptome, which bears signatures of both proliferation/cell survival and apoptosis/cell death.

## Results and Discussion

### Experimental Design

Given the difficulties in obtaining sufficient primary human lung epithelial cells to study the complex response to asbestos, many studies have instead employed the A549 human adenocarcinoma cell line. In doing so one must keep in mind that the transformed cell line may not be entirely applicable to normal human lung cells. However, all processes noted below are recapitulated in primary human lung epithelial cells, suggesting that A549 cells represent a valid model system. Importantly, use of a human cell line avoids the inherent differences that are seen between individual patients. Alveolar epithelial type II cells are key participants in inflammation, fibrogenesis, and carcinogenesis [23] and have been described as the targets of asbestos-associated lung carcinomas [24].

The conditions used for obtaining the gene expression profile for crocidolite treated A549 cells were chosen to mimic conditions where a variety of biochemical observations have previously been made in our laboratories. Exposure of A549 cells to 6 μg/cm^2 ^crocidolite for 24 hours had previously been shown to result in the α_v_β_5 _integrin receptor-mediated endocytosis of asbestos fibers [25], mobilization of iron from the fibers within the cell [26], upregulation of ferritin protein to combat the iron overload [27], production of reactive oxygen intermediates [28], efflux of reduced glutathione [9], DNA damage [7], PARP cleavage and activation of initiator caspases [29]. Thus, at 6 μg/cm^2 ^of crocidolite the microarray results could be related to an extensive body of biochemical information already available in A549 cells. In addition, this cell line has been used by other investigators for several studies to explore the mechanisms of DNA damage [30], apoptosis [10], and invasiveness [31]. Use of the A549 human lung epithelial cell line complements studies done in mesothelial cells [32] and by the less carcinogenic chrysotile in bronchial epithelial cells [33]. Our goal was to extend our results beyond lists of genes and ontological classifications to discover new pathways based on functional interactions. Rather than diffusing statistical power by examining differences among multiple time points and asbestos concentrations, this experiment focused on the use of chemically defined crocidolite asbestos, a single concentration of crocidolite versus a control condition, and a single exposure time. This focus allowed for three replicates of mRNA from control and crocidolite-treated cells to be analyzed using the Affymetrix HGU133 Plus 2.0 GeneChip to provide the most comprehensive whole genome expression profile.

### Analysis of the Microarray Data

**Figure 1:**
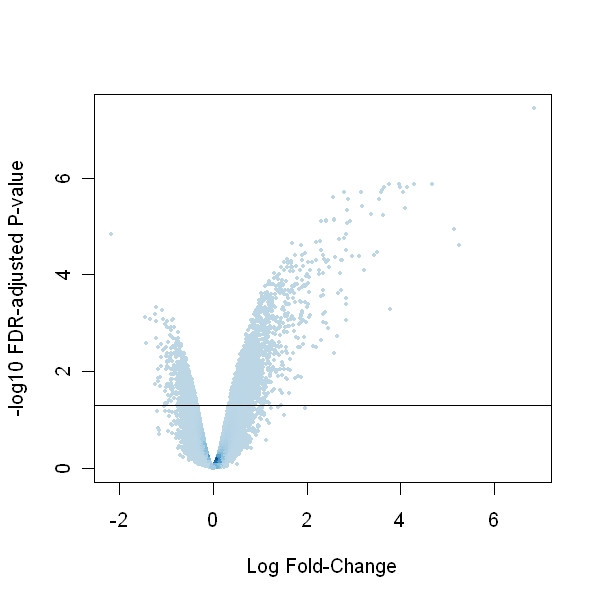
**Volcano plot showing the magnitude of differential expression (log2 fold-change) compared to the measure of statistical significance (-log10 q-value). **Color is on the density scale, so darker colors indicate over-plotting of points. Statistically significant genes are observed above the horizontal line, which corresponds to a q-value of 0.05. The low variability in the data caused a large number of genes (2,546) to be classified as statistically significant.

Graphical checks of the gene expression data revealed a high-quality data set, with no spatial artifacts in the chip images, and a high degree of reproducibility within both the control and treated replicates (Additional File 1). Using tools described in the Methods Section and a false discovery rate [34] of 0.05, 2,546 genes with q-values (FDR-adjusted p-values) less than 0.05 were called statistically significant. These results are represented in a volcano plot (Figure 1).

### Crocidolite Induces Large Changes in the Transcriptome of A549 Human Lung Epithelial Cells

Among the 54,120 probe sets on the GeneChip, 1808 were significantly up-regulated (492 of which were two-fold or greater), and 738 were significantly down-regulated (27 of which were two-fold or greater) when A549 cells were exposed to 6 μg/cm^2 ^crocidolite asbestos for 24 h. Of the probe-sets that changed two-fold or greater, 234 correspond to known genes for the up-regulated probe sets, and 16 of the down-regulated probe sets correspond to known genes (some genes were represented more than once on the chip). Genes that increased in expression five-fold or greater or decreased two-fold or greater and were associated with q-values less than 0.05 are shown in Table 1. A complete table of all significant genes can be found as Additional File 2. The observation that most of the expression changes are upregulated is contradictory to a previous microarray study in A549 cells [22] which used a smaller dose of crocidolite (2 μg/cm^2^). A direct comparison of the two data sets was difficult due to differences in experimental design. Namely, replicates at a single time point were used in the current study versus single chips over a time course, and a lower crocidolite concentration, which is a likely cause for some differences. Cells exposed to asbestos may demonstrate a hierarchical oxidative stress response [35]. Additionally, small amounts of asbestos have been shown to result in proliferation [24]. This may be attributed to a transient response to an increase in iron, which is limiting in cells in culture. However, comparison of individual expression changes in our data set to other known experimental results (discussed below) demonstrated that our data set is consistent with the literature.

**Table 1:** List of genes for which expression increased five-fold or greater or decreased two-fold or greater

**Fold Change**	**Gene Symbol**	**Gene Name**
115.7	**EGR1**	Early growth response 1
38.3	FOS	v-Fos FBJ murine osteosarcoma viral oncogene homolog
25.3	ATF3	Activating transcription factor 3
19.5	**GEM**	GTP binding protein overexpressed in skeletal muscle
17.1	**NR4A2**	Nuclear receptor subfamily 4, group A, member 2
16.6	IL8	Interleukin 8
15.9	FST	Follistatin
15.6	PPP1R15A	Protein phosphatase 1, regulatory (inhibitor) subunit 15A
13.7	**STC1**	Stanniocalcin 1
12.1	**MAFF**	v-Maf musculoaponeurotic fibrosarcoma oncogene homolog F (avian)
11.2	**CXCL2**	Chemokine (C-X-C motif) ligand 2
10.4	**TNFAIP3**	Tumor necrosis factor, alpha-induced protein 3
9.4	**MXD1**	MAX dimerization protein 1
9.1	**ARID5B**	AT rich interactive domain 5B (MRF1-like)
9.0	**NR4A3**	Nuclear receptor subfamily 4, group A, member 3
8.6	**JUN**	v-Jun sarcoma virus 17 oncogene homolog (avian)
7.8	**LOC153222**	adult retina protein
7.4	**BRE**	brain and reproductive organ-expressed (TNFRSF1A modulator)
7.2	**MCTP1**	Multiple C2 domains, transmembrane 1
7.2	**DDIT3**	DNA-damage-inducible transcript 3
7.2	**FOSB**	FBJ murine osteosarcoma viral oncogene homolog B
7.1	GDF15	Growth differentiation factor 15
6.9	**CITED2**	Cbp/p300-interacting transactivator, with Glu/Asp-rich carboxy-terminal domain, 2
6.9	**DUSP6**	Dual specificity phosphatase 6
6.6	**CXCL1**	Chemokine (C-X-C motif) ligand 1 (melanoma growth stimulating activity, alpha)
6.5	**IL24**	Interleukin 24
6.3	**SPRY2**	Sprouty homolog 2 (Drosophila)
6.1	**PTGS2**	Prostaglandin-endoperoxide synthase 2 (prostaglandin G/H synthase and cyclooxygenase)
6.0	**KLF6**	Kruppel-like factor 6
6.0	**IL11**	Interleukin 11
5.6	**IRAK2**	Interleukin-1 receptor-associated kinase 2
5.5	**IL6**	Interleukin 6 (interferon, beta 2)
5.5	**HIST1H4H**	Histone 1, H4h
5.4	**FNIP1**	folliculin interacting protein 1
5.3	**HIST2H2BE**	Histone 2, H2be
5.2	**MCL1**	Myeloid cell leukemia sequence 1 (BCL2-related)
5.1	**DUSP10**	Dual specificity phosphatase 10
5.1	**DHRS2**	Dehydrogenase/reductase (SDR family) member 2
5.1	**SYNE1**	Spectrin repeat containing, nuclear envelope 1
5.1	**HAS2**	Hyaluronan synthase 2
5.1	**PER1**	period homolog 1 (Drosophila)
5.1	**ZBTB10**	zinc finger and BTB domain containing 10
-2.0	**FN1**	Fibronectin 1
-2.0	**BCL2L11**	BCL2-like 11 (apoptosis facilitator)
-2.0	**COL5A1**	collagen, type V, alpha 1
-2.0	**VAV3**	vav 3 oncogene
-2.0	**CSRP2BP**	CSRP2 binding protein
-2.0	**PRSS23**	protease, serine, 23
-2.1	**NRP2**	Neuropilin 2
-2.1	**DAPK1**	Death-associated protein kinase 1
-2.1	**CDH1**	Cadherin 1, type 1, E-cadherin (epithelial)
-2.2	**ST8SIA4**	ST8 alpha-N-acetyl-neuraminide alpha-2,8-sialyltransferase 4
-2.2	**RNASE4**	Ribonuclease, RNase A family, 4
-2.2	**FGG**	Fibrinogen gamma chain
-2.1	**STAT4**	Signal transducer and activator of transcription 4
-2.1	**NTRK3**	Neurotrophic tyrosine kinase, receptor, type 3
-2.3	**PCDH9**	Protocadherin 9
-2.2	**CYP4F3**	Cytochrome P450, family 4, subfamily F, polypeptide 3
-2.2	ST8SIA4	ST8 alpha-N-acetyl-neuraminide alpha-2,8-sialyltransferase 4
-2.3	**MAP2K6**	Mitogen-activated protein kinase kinase 6
-2.3	**LXN**	latexin
-2.3	**TM4SF20**	transmembrane 4 L six family member 20
-2.4	**AKR1C1///AKR1C2**	Aldo-keto reductase family 1, member C1 (dihydrodiol dehydrogenase 1; 20-alpha (3-alpha)-hydroxysteroid dehydrogenase)///aldo-keto reductase family 1, member C2 (dihydrodiol dehydrogenase 2; bile acid binding protein; 3-alpha hydroxysteroid dehydrogenase, type III)
-2.7	**SLC40A1**	Solute carrier family 40 (iron-regulated transporter), member 1

A549 human lung epithelial cells were exposed to crocidolite asbestos at 6 g/cm^2 ^for 24 h. Genes for which expression levels increased 5.0 fold or greater, or decreased 2.0 fold or greater, having q values less than 0.05 are shown. Probe sets associated with products of unknown function were not included. Bold-faced genes were not observed in the statistically upregulated expression profile of hydrogen-peroxide-treated A549 cells [36]. A list of all genes for which expression levels changed 2.0 fold or greater can be found as Additional File 2.

**Figure 2:**
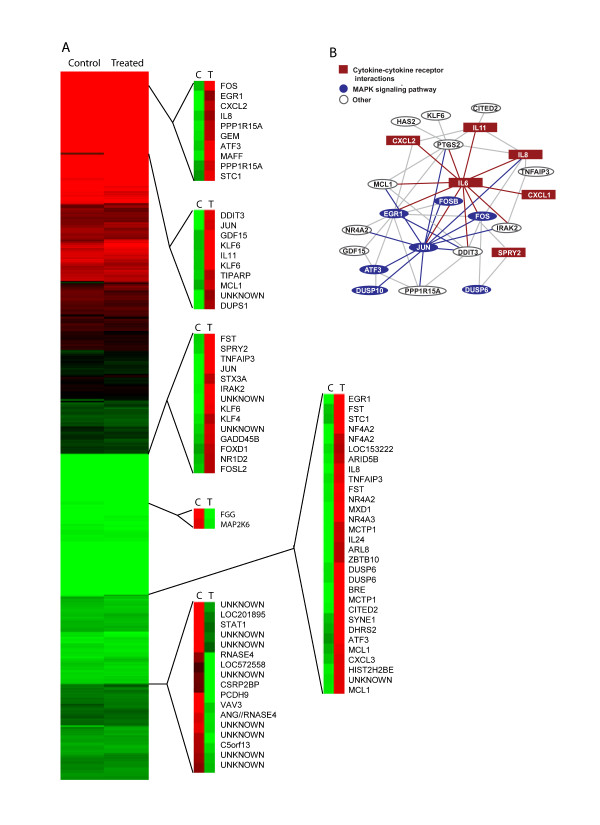
**Unsupervised hierarchical clustering analysis of A549 probe sets in which the expression was altered by crocidolite.(A) **The main dendogram represents the intensity of each probe set in relation to the entire data set with green being low and red being high. Four major clusters of upregulated genes and two clusters of downregulated genes were observed and are shown as bracketed dendograms where the color represents the intensity of each probe set in relation to each specific cluster. **(B) **STRING analysis using the genes within the upregulated clusters demonstrated a functional relationship between 24 of the genes which encompassed cytokine, MAPkinase and JNK/SAPK signaling pathways.

Hierarchical clustering analysis (Figure 2A) of our data set showed that the majority of the up-regulated probe sets clustered together in four different hierarchical clusters. The down-regulated probe sets were mainly in one hierarchical cluster, but had several individual genes spread throughout the dendrogram. Within the four up-regulated clusters, 24 genes that were upregulated 5-fold could be mapped by functional String analysis shown in Fig 2B. Most of the gene products are connected; i.e., could be linked functionally by way of known molecular interactions. Three major pathways (MAPK, JNK/SAPK and cytokine-cytokine receptor interactions) are represented in the cluster. This result is consistent with what is currently known about the effect of asbestos in epithelial and mesothelial cells. When the down-regulated genes were functionally clustered, only two (*fyr *and *uvo*) out of 16 had a connection (data not shown).

### Validation of the Microarray Data

Validation of the microarray data was confirmed by quantitative RT-PCR on four of the genes (*Egr1*, *ATF3*, c-*Jun*, and *JunB*) and was further corroborated with previously noted results from experiments using human cells/cell lines (Table 2). Quantitative RT-PCR also demonstrated that increases in Egr-1, ATF3, c-Jun, and JunB message occurred after 6 hours and continued to increase through 24 hours (data not shown). Increased mRNA expression from *ATF3*, c-*Jun*, and *JunB *was also observed by quantitative RT-PCR in primary human epithelial cells from small airways (SAEC) treated with crocidolite (data not shown). Increased protein expression in A549 cells exposed to crocidolite was observed for ATF3, c-Jun, and JunB (data not shown). Finally, previous results using asbestos-treated A549 cells have demonstrated increased mRNA and/or protein levels for *IL8, TP53, HMOX1, CDKN1A, SOD2 *and c-*myc *(see Table 2 for additional references). The reproducibility of the microarray replicates, the significance level of the expression changes, and the validation by quantitative RT-PCR suggest that a highly useful and validated data set was obtained in this study. Furthermore, the large number of significant genes allowed us to perform a comprehensive analysis of the crocidolite-induced transcriptome in human lung epithelial cells.

**Table 2:** Validation of the microarray data from crocidolite-treated human A549 cells

**Gene**	**Alias for Protein Product**	Affymetrix* (fold change) this study	**QRT-PCR (fold change) this study**	**Changes in mRNA levels in human cells observed by other groups**
*EGR1*	Egr1	115.7	281.7 ± 3.8	
*ATF3*	ATF3	25.3	23.7 ± 0.1	
c-*Jun*	c-Jun	8.6	10.5 ± 0.7	
*JunB*	JunB	2.2	3.6 ± 0.2	
*IL8*	Interleukin 8	16.6	N.D.	Increased [73]
*TP53*	p53	1.8	N.D.	Increased [16,74]
*SOD2*	Mn-SOD	2.2	N.D.	Increased [75]^‡^
*HMOX1*	Heme oxygenase 1	2.1	N.D.	Increased [75,76]^†^
c-*myc*	myc	2.0	N.D.	Increased [77]
*CDKN1A*	p21, Cip1	1.9	N.D.	Increased [16,74]

*represents significantly greater than control, q less than 0.05; N.D., not determined

^† ^chrysotile-treated A549 human lung epithelial cells

^‡ ^crocidolite-treated human pleural mesothelial cells

### Specific Changes in the Crocidolite-Treated Transcriptome

Although it is clearly established that asbestos induces DNA damage in both primary cells and cell culture lines, the literature illustrates that the molecular mechanism underlying this process and how the fate of the cell is dictated are multifaceted. Although ROS are intimately tied to the mechanism of asbestos-induced fibrosis and carcinogenesis, ROS alone do not offer a complete understanding of the asbestos response. In order to identify genes for which expression level changes are specific for crocidolite compared to oxidative stress, we compared our data set to expression changes that occur in A549 cells when exposed to hydrogen peroxide. In a previous study that documented changes in the A549 cell transcriptome, Cotgreave and co-workers [36] showed that treatment of A549 cells with hydrogen peroxide results in DNA damage and apoptosis. Both hydrogen peroxide and crocidolite asbestos induced the upregulation of *TNFRS10B, PPP1R15A, GADD34, CDKN1A, BTG2, DUSP1, DUSP5, DUSP14, SDC4, GDF15, IL8, ADM, FST, IER3, FOS, HMOX1, ATF3*, and *ZFP36*. Therefore, expression changes in these genes may represent a response to an oxidative stress. Several unique genes that are differentially regulated in the crocidolite data set are noted in Table 1 and include some genes which are associated with carcinogenesis such as *STC1, IL6, FN1, BRE*, and *PTGS2*. Differences between the hydrogen peroxide-treated and crocidolite-treated transcriptomes may be due to the additional iron released from fibers, present in cells treated with crocidolite, or reactive nitrogen species, changes in glutathione content and/or the involvement of processes initiated at the cell surface by the fiber. It will be very interesting to investigate if these apparent crocidolite-specific gene regulations play a role in crocidolite-induced cytobiological endpoints.

### Ontological Analysis of the Crocidolite-Treated Lung Transcriptome

**Figure 3:**
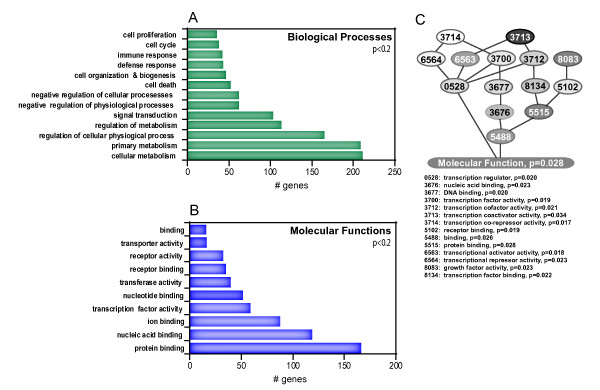
**Gene ontology analysis of the (**A**) Biological Processes and (**B**) Molecular Functions most affected by crocidolite in A549 cells.** In (**C**) a global test to identify ontologies related to transcription was used to pinpoint significant functions. The p-values for each of the GO terms (abbreviated as the last four digits of each ontology's identifier) has been overlaid onto the hierarchical tree where the darkest gray node represents a p = 0.034 and the lightest represents a p = 0.017.

In order to identify themes in the global expression pattern of crocidolite-treated A549 cells, we used Gene Ontology (GO) classification. The differentially expressed genes (up-regulated/down-regulated ± 2-fold with p-values less than 0.2) were analyzed using Onto-Express . The Biological Process tree was expanded to the fourth tier in the hierarchy as a balance between specificity and coverage. Processes affected by crocidolite are shown in Figure 3A. Within the Cellular Metabolism classification, processes involved in transcription and phosphorus metabolism were highly represented. Expansion of Molecular Functions to the third tier revealed a large number of genes with functions related to transcription (over-represented in Protein binding (Figure 3B)). This observation was expanded upon by performing a global test [37-39] to identify specific gene ontologies related to transcription, where the expression levels of all the genes in the ontology give useful information for predicting the clinical outcome (or in this case, a cellular outcome of crocidolite exposure). Ontologies of particular interest related to transcription factor activity (GO:0003700) are summarized in Figure 3C, with nodes colored to correspond to global test p-values; the lighter nodes have lower p-values. To reduce visual clutter, only the last four digits of each ontology's identifier are reported in Figure 3C. The ontological analysis illustrates that transcriptional corepressor activity and repressor activity are highly represented in the data set. Overall the transcriptome of crocidolite-treated human lung epithelial (A549) cells is heavily populated by gene products associated with transcription.

We also noted that DNA repair terms were not highly represented in the data set. Although this result was initially surprising, we noted that the transcriptome of A549 cells exposed to hydrogen peroxide, which also induces DNA damage, was also underrepresented in DNA repair terms [36]. In order to gain a better understanding of how the transcriptome of crocidolite-treated A549 cells is reorganized in response to the resulting DNA damage, the results of the global test discussed above were also applied to ontologies related to DNA damage/repair. Although this analysis (see Additional File 3) confirms the overall observation that many of the processes related to DNA repair/damage are not highly represented, the analysis also highlights specific nodes in the GO tree related to DNA damage-induced phosphorylation (GO:6975), DNA dealkylation (GO:6307), DNA methylation (GO:6306), and DNA alkylation (GO:6305). In light of the several studies directed at linking promoter methylation status and carcinogenesis (reviewed in [40]), it is interesting to point out that in as little as 24 hours of crocidolite exposure, the A549 transcriptome may be poised to affect the DNA methylation status that is associated with lung cancer pathogenesis.

### Identification of Novel, Putative Crocidolite-Related Genes

Even though this study was initiated to understand the broad changes that occur in the lung transcriptome upon crocidolite exposure, we also sought to identify novel genes that had no previous association with crocidolite and to identify genes that may be downstream targets of well-known crocidolite-related players. Given the complex effects that asbestos has on the lung system, we expected that a systems biology approach may provide novel avenues to study. *NR4A1*, *NR4A2*, and *NR4A3 *belong to the steroid nuclear hormone receptor superfamily of immediate early genes that are induced by serum, growth factors and receptor engagement and are thus implicated in cell mitogenic responses. Although previously characterized as pro-survival, studies have also suggested an important role for these receptors in cell transformation and tumorigenicity via their anti-apoptotic and pro-apoptotic functions [41]. Thus, depending on cellular context, these gene products may serve as switches in determining cell fate. All three members of this family show increased expression in our data set (Additional File 2) but have not previously been implicated in crocidolite-induced pulmonary toxicity.

Modulation of apoptosis can also be affected by BRE (brain and reproductive organ-expressed protein), a stress-modulating protein also known as TNFRSF1A modulator. *BRE *expression was upregulated >7-fold in crocidolite-treated A549 cells. Exogenous overexpression of BRE can attenuate intrinsic apoptosis and promote growth of the transfected Lewis lung carcinoma line in mice [42] which is consistent with the recent finding that BRE protein is overexpressed in human hepatocellular carcinomas [43]. Given the ability of BRE to interact with both Fas [44] and the TNF receptor 1 [45], and the observation that TNFα can attenuate asbestos cytotoxicity in mesothelial cells [17], it will be very interesting to investigate possible roles of BRE in crocidolite-treated human lung cells. Also noteworthy is the upregulation of several "early response" NF-κB targets [46] in our dataset (Additional File 2) including *TNFAIP3, IL8, IL6, CXCL1, CXCL2, CXCL3, PTGS2*, and *PLAU*. Activation of the NF-κB pathway is thought to play a critical role in cell survival in asbestos-treated cells [17], but only a few of the downstream targets of NF-κB have been identified.

The relationship between asbestos and calcium has received little attention in recent years, but the initial studies suggest that calcium may have an important role to play (reviewed in [47]). Perhaps most compelling is the ability of the calcium-chelator Quin-2 to prevent crocidolite-induced DNA breaks [48]. Additionally, several players in the asbestos response are regulated by calcium levels, e.g., protein kinase C [15]. We were therefore interested in determining if the crocidolite-treated transcriptome demonstrated any clues regarding the regulation of calcium homeostasis. We observed that expression of *MCTP1 *and *STC1 *were both upregulated in our data set by 7.3- and 12.9-fold, respectively. MCTP1 is a transmembrane protein that binds calcium ions via C_2 _domains. Unique properties of this protein suggest that these proteins function in Ca^2+ ^signaling at the membrane [49]. Stanniocalcin 1 (STC1) has long been studied as a regulator of both phosphate and calcium homeostasis in bony fish, but has recently received attention in mammalian systems. STC1 is a glycoprotein present in a variety of mammalian tissues where it can function as a regulator of gene expression and modulator of transendothelial cell migration [50], and can also affect cellular metabolism by perturbing mitochondrial electron transport chain and mitochondrial calcium transport [51]. STC1 affects calcium homeostasis in the heart [52,53] and the brain [54]. Growing evidence also points to a correlation between STC1 expression and the development of human cancers [55,56]. Quantitative RT-PCR also demonstrated a 30.7 ± 5.1-fold increase in STC1 message in primary human SAEC exposed to crocidolite.

Finally, in recent years a significant number of studies have been directed at understanding how the disruption of dynamic chromatin remodeling is linked to carcinogenesis. Mechanisms including the previously mentioned DNA methylation status and the use of histone modifications have led to the discovery of prognostic biomarkers [57] and the use of HDAC inhibitors as cancer therapeutics [58]. It is of little surprise then to find several differentially regulated genes in crocidolite-treated A549 cells that could participate in chromatin remodeling including the Jumonji domain histone demethylases *JMJD1C*, *JMJD3 *and *JMJD1A*, all of which showed increased expression (Supplementary Table 1). Other genes of interest are discussed below.

### Pathway Analysis Provides Unique View of Function-Based Networks in Crocidolite-Treated Cells

In order to extract novel biological insight from the large number of genes upregulated/downregulated in our study, we employed a structured network knowledge-based approach to analyze genome-wide transcriptional responses in the context of known functional interrelationships among proteins, small molecules and phenotypes . Networks are generated not only based on functional interactions but also statistical likelihood. Genes/gene products are represented as nodes and are color-coded to represent fold-change in expression level. Interactions between nodes are designated as edges, or lines, and represent physical, transcriptional, and enzymatic interactions.

#### Statistically Significant Function-based Networks and Pathways

**Figure 4:**
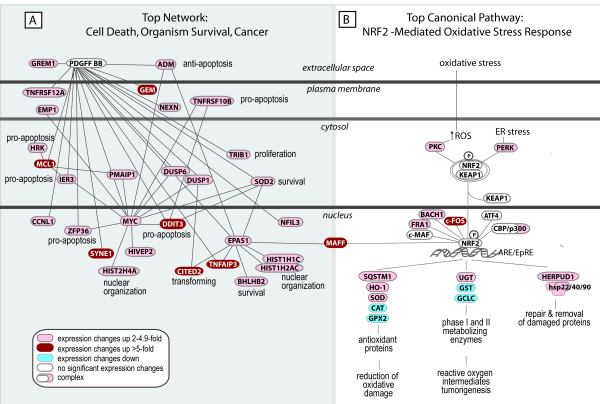
**Spatial depiction of the top scoring network and canonical pathways detected in crocidolite-treated A549 cells by Ingenuity Pathway^® ^Analysis.** The network was algorithmically generated based on the connectivity of each of the transcripts and the molecular interaction knowledge base. Each node represents a gene or gene product for which mRNA expression was upregulated (red) or downregulated (blue) in crocidolite-treated A549 cells. Edges/lines connecting the nodes represent molecular interactions between genes and/or gene products and are supported by at least one reference from the literature, a textbook, or from canonical pathway information stored in the Ingenuity Pathways Knowledge Base. The Nrf2-mediated oxidative stress canonical pathway identified by Pathway Analysis software shows differential upregulation of select genes within the cytoprotective arsenal.

Using expression changes that were differentially regulated by ± 2-fold and having p values less than or equal to 0.01, Ingenuity Pathway^® ^Analysis demonstrated a highly complex set of 16 interconnected networks. These networks were related to cell death, cancer, cell cycle, cellular growth and proliferation, and gene expression. All networks had at least one gene in common and many had two genes in common, underscoring the interplay and complexity of the crocidolite response. The top scoring network (Figure 4A) was composed of genes related to cell death, organism survival and cancer, and highlighted *MYC*, *PDGF BB*, and *EPAS1/HIF2 *as prominent nodes. In particular, *EPAS1 *was a node for four genes whose expression levels changed 6-fold or greater. EPAS1 (endothelial PAS domain protein-1, or HIF2α) is one of the transcription factors which belong to the basic helix-loop-helix PAS family. It shares sequence similarity to HIF-1α and analogously to HIF-1α, regulates transcription of *VEGF *and is observed to be upregulated at both the message and protein levels in A549 cells as a result of hypoxia [59]. Prognostic significance of increased levels of EPAS1 mRNA and/or protein has been observed in liver [60] and colon [61] cancers. Interestingly, EPAS1 -/- mice show impaired reactive oxygen species homeostasis [62], which may be linked to the role of EPAS1 in maintaining mitochondrial homeostasis [63], creating a hypothetical link between EPAS1 function and response to crocidolite.

This first network (Figure 4A) is linked to the Nrf2-mediated oxidative stress pathway (one of the top scoring canonical pathways in our data set shown in Figure 4B) via the *MAFF *node. Cellular use of this canonical pathway has been linked to tumor cell survival by maintaining cellular redox homeostasis and protection against oxidative insult. Heterozygous Nrf2 (+/-) mice exposed to crocidolite fibers exhibit accelerated development of malignant mesotheliomas compared to wild-type littermates [64]. Several of the known Nrf2 targets did not show significant changes in expression levels in our data set (data not shown). Although not all of the downstream targets of *NRF2 *were differentially upregulated upon exposure to crocidolite at 6 μg/cm^2^, message levels for many of the Nrf2 targets were present in above average amounts on both the control and treated chips. Furthermore, data suggests that this pathway may be activated in a hierarchical fashion [35], dependent on exposure. Selective activation of the Nrf2 pathway may contribute to carcinogenicity of the crocidolite fibers, while dysfunctional constitutive activation of Nrf2 [65] has been observed in non-small-cell lung cancer.

The topscoring network illustrates the interplay between processes involved in both cell survival/proliferation and cell death/apoptosis when A549 cells are exposed to crocidolite. Activation of the Nrf2 cytoprotective pathway in the transcriptome of crocidolite-treated A549 cells is imperfect. The observed selective Nrf2 target expression would define an environment that is rich in hydrogen peroxide and incapable of redox homeostasis.

#### A Global View of the Functional Networks in A549 Cells in Response to Crocidolite

**Figure 5:**
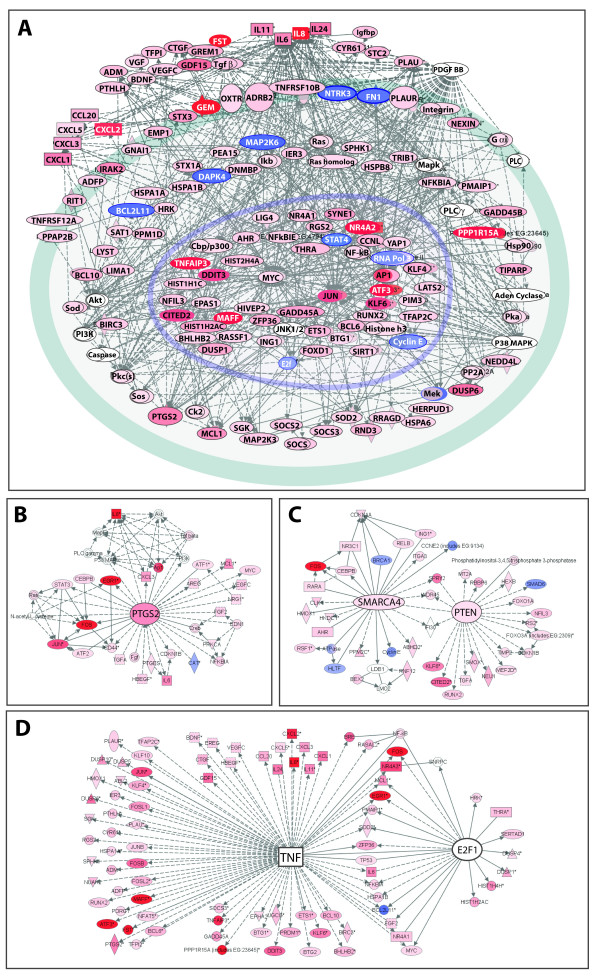
**Pathway analysis of representative genes involved in the response of A549 cells exposed to crocidolite.** In **(A) **the top five scoring networks were merged to create a cellular model consisting of ~150 genes for which expression levels changed ± 2-fold and demonstrated p-values less than 0.01. Nodes representing genes are colored red for upregulation or blue for downregulation and the intensity of the color reflects the degree of up- or downregulation. Lines connecting the nodes are indicators of interactions found in the knowledge database or current literature. In (**B**) and **(C) **the networks surrounding *PTGS2*, *SMARCA4*, and *PTEN *have been expanded for clarity. In **(D**) the relationship between *E2F1 *and *TNF *is observed.

Another approach to gain insight about the functional significance of the global changes in crocidolite-induced gene expression is to merge the individual networks and then identify nodes that are used frequently. Over 500 nodes were observed within the 16 networks, making presentation of the global cellular network difficult. Instead, the top five networks were merged using all known interactions in the knowledge-based database and the nodes arranged according to subcellular location resulting in Figure 5A. This representation comprises ~150 genes and their interactions and illustrates a transcriptome encoding an array of extracellular growth factors and cytokines. Even though a population of exposed cells undergoes apoptosis, some cells may survive and be stimulated to proliferate based on factors released from neighboring cells. Several high impact nodes including *MYC, JUN*, Akt, p38, and *PDGF BB*, all of which are established players in the response to asbestos, are also present. Other genes that formed prominent nodes in the prototypical cell or adjacent networks included *PTGS2*, *SMARCA4*, *PTEN*, and *E2F1*. They are shown as separate networks for clarity in Figures 5B, C and 5D. The *PTGS2 *product, Cox-2, PTEN and E2F1 have previously been implicated in carcinogenesis, but their roles in the epithelial cell response to asbestos have not been studied. Brg1, the protein product of *SMARCA4*, is a SWI/SNF related chromatin remodeling factor which recognizes acetylated lysine groups through bromo domains and is involved in cell growth arrest and apoptosis. Oxidative stress and TNF-α induce histone acetylation and NF-κB/AP-1 activation in alveolar epithelial cells [66] suggesting a potential mechanism to alter gene transcription in lung inflammation using Brg1. Inspection of the network surrounding the *E2F1 *node identified several genes whose expression was differentially regulated by asbestos that also demonstrated molecular interactions with *TNF*. Given the relationship between TNF and NF-κB activation observed in mesothelial cells exposed to crocidolite [17], and the recent identification of E2F1 as a transcriptional activator recruited by NF-κB [67], investigation into the role that E2F1 plays in human lung epithelial cells exposed to crocidolite should be forthcoming.

This analysis has provided the first function-based global view of the crocidolite-treated A549 transcriptome. Several new candidate crocidolite-related genes were identified in the context of experimentally observed findings. Apparent from the global analysis is a transcriptome bearing signatures of both apoptosis/cell death and cell survival/proliferation.

#### Using Pathway Analysis to Probe the Role of p53

**Figure 6:**
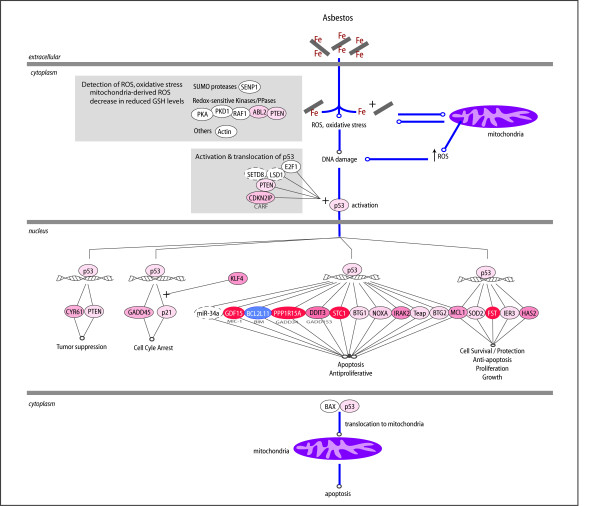
**Asbestos induces a p53-mediated response in A549 lung cells. **Experimental data is indicated by the thick blue edges and is discussed in more detail in the text. Known p53 targets which may have a role in the asbestos response and their biological effects are shown using thin gray lines. Nodes are color coded shades of red when expression levels increased and shades of blue when expression levels decreased. Nodes surrounded by dashed lines represent genes/gene products which were not represented on the gene chip but which displayed a functional relationship to queried nodes.

Pathway Analysis also detected significance in p53-mediated processes. This result is consistent with the observation that both amosite and crocidolite induce p53 activation. Specifically, Kamp and co-workers who showed that p53 mediates amosite-induced apoptosis through mitochondrial dysfunction in A549 cells [10]. Although the mechanism is not clear, ROS generated by the mitochondria, p53-mediated transcription, and translocation of p53/BAX appear to be necessary components for apoptosis to occur. The authors suggested that other pro-apoptotic pathways may also be activated and a more thorough study of p53-targeted transcription pathways was needed. Figure 6 shows a scheme representing these findings overlaid with the expression changes from our data set and molecular interactions identified through network analysis. Since it is unknown whether ROS detection and p53 activation require new protein synthesis, we did not restrict this part of the analysis to differentially regulated genes. Several genes whose expression depends on p53 could be identified that were associated with both apoptosis and proliferation or survival. These results may reflect the expected heterogeneity in the population of cells after exposure to crocidolite, as discussed previously. Although crocidolite induces apoptosis in many of the cells, the bulk transcriptome shows evidence that transcripts are present which could tip the balance in favor of cell cycle arrest and/or cell survival or proliferation. Message levels of the p53-induced molecular switch p21^CIP/WAF1 ^and KLF4 were upregulated in crocidolite-treated cells. Together these two products can have an anti-apoptotic effect [68].

The importance of p53 in mediating apoptosis in asbestos-treated A549 cells has been documented [10]. Analysis of p53 targets upregulated in the crocidolite-treated A549 transcriptome identified several candidate genes that may function in the observed apoptosis. Interestingly, we also identified several p53 targets associated with cell survival/proliferation. This suggests that p53 may be an important molecular turning point in the decision of crocidolite-treated cells to undergo apoptosis or to proliferate, even in the presence of damaged DNA.

## Conclusion

In this study, we have provided a statistically robust and comprehensive global gene expression profile of A549 human lung epithelial cells exposed to crocidolite asbestos. Our data reveal a much altered transcriptome in which a large number of genes show upregulated expression. Crocidolite-treated A549 cells are rich in transcripts encoding extracellular growth factors and cytokines and intracellular regulators/mediators of transcription. A global view based on functional molecular interactions illustrated an intricate network of paths associated with both apoptosis and proliferation/cell survival. This network allowed for the identification of novel, putative crocidolite-related genes, leading to several new hypotheses regarding genes which are important in the response of human lung epithelial cells to crocidolite.

Our analysis demonstrates the power of a functional network genomics approach to 1) identify and explore relationships between genes of known importance 2) identify novel candidate genes, and 3) observe the complex interplay between genes/gene products that function in seemingly different processes. This study represents the first function-based global approach toward understanding the response of human lung epithelial cells to the carcinogen crocidolite. We have provided graphical representations of the highly interconnected networks that will be instrumental in modeling the impact of new research findings. Our global function-based approach introduces new insights and novel avenues which can now be investigated in more detail to understand the effects of crocidolite on the human lung.

## Methods

### Reagents and Antibodies

Crocidolite was obtained from Dr. Richard Griesemer, NIEHS/NTP. The chemical formula for crocidolite is Na_2_Fe^III^_2_(Fe^II^, Mg)_3_Si_8_O_22_(OH)_2_. It has a mean length of 10 μm, a density of 3.2–3.3 g/cm^3 ^and contains 27% iron by weight [69]. A custom prepared Ham's F-12 cell culture medium free of iron salts and 0.5% trypsin with 0.2% EDTA were obtained from Invitrogen Inc. (Carlsbad, CA). Fetal bovine serum (FBS) was purchased from Hyclone (Logan, UT). Antibodies for Egr-1 (sc-189) and ATF3 (sc-188) were purchased from Santa Cruz Biotechnology (Santa Cruz, CA) and the antibody for β-actin was purchased from Sigma (St. Louis, MO). Anti-rabbit and anti-mouse enzyme-conjugated secondary antibodies were purchased from Jackson Immunoresearch Laboratories Inc. (West Grove, PA). Primers were purchased from IdtDNA (Coralville, IA). All remaining reagents were purchased in the highest purity possible.

### Cell Culture and Treatment

A549 cells were purchased from American Type Culture Collection and grown in F12 medium without iron, with 10% FBS and 50 μg/mL gentamicin (BioWhittaker, Walkersville, MD). Cells were plated for treatments at a concentration of 20,000 cells/cm^2 ^and incubated 24 h before treatment. For all treatments, medium was removed and replaced with either fresh medium (for control samples) or medium containing the appropriate stimulus (treated samples). For crocidolite treatments, fibers were suspended in sodium bicarbonate at a concentration of 1 mg/mL and immediately diluted to a final concentration of 6 μg/cm^2 ^in complete medium. Sodium bicarbonate is used specifically to avoid iron mobilization in the media before the fibers can by endocytosed and maintains the pH [26]. These are the exact same conditions and methodologies used previously in several other studies [9,11,25-27].

### RNA Extraction and Use of DNA Microarrays

mRNA was isolated from cells at passage 9 using TRIzol^® ^reagent from Invitrogen (Carlsbad, CA) following manufactures directions. This procedure yielded RNA of both high quantity and quality, as verified by both the A_260_/A_280 _ratio (> 1.8) and the Agilent 2100 Bioanalyzer (Agilent Technologies, Palo Alto, CA). The extracted RNA was processed by the Affymetrix core service at the Center for Integrated BioSystems, Utah State University (CIB-USU; Logan, UT). RNA was processed as per the manufacturer's instructions (Affymetrix Inc., Santa Clara, CA) for the first-strand cDNA synthesis and amplification, followed by cDNA synthesis and labeling. The cDNA was hybridized overnight to the HGU133-Plus2.0 Human Genome 2.0 genechips and post-hybridization washing, detection and data collection were performed as per manufacturer's instructions. Three biological replicates were performed. Each replicate consisted of a control plate and a crocidolite-treated plate. All experiments were done at passage 9.

### Statistical Analyses

Using tools from the Bioconductor project [70], the data were pre-processed using the RMA algorithm [71], and a test for differential expression between control and treated (crocidolite-exposed) conditions was performed using the moderated t-statistic from the limma/eBayes approach [72]. The raw p-values from this test were adjusted to control the false discovery rate [34] at 0.05. Dendrograms (Figure 2) were drawn by Hierarchical Clustering Explorer (HCE; ) software version 3.0 to summarize RMA-normalized data after averaging across replicates. The cutoff value for minimum levels of gene expression was based on the whole genome expression profiles generated by HCE. The median cutoff value was set at the software-calculated median of gene expression and was colored black. The data were further subdivided into gene functional classes that had been annotated by the Human Genome Database. Each class was then clustered separately and the determined cutoff values were applied to generate colored expression maps for each individual class for ease of visualization. In addition, a global test [37-39] was used to identify gene ontologies of interest, where the expression levels of the genes in the ontology were statistically significant (p-value less than 0.05) for predicting the clinical outcome (crocidolite exposure). STRING analysis was performed at  using default parameters.

### Quantitative RT-PCR

mRNA was isolated using TRIzol^® ^reagent from Invitrogen (Carlsbad, CA) following manufactures directions. DNase from the RNAqueous^®^-4PCR kit (Ambion Inc., Austin, TX) was added to eliminate genomic DNA and removed following the manufacture protocol. cDNA was transcribed using SuperScript™ II Reverse Transcriptase (Invitrogen Inc., Carlsbad, CA). Specificity was confirmed by performing a melting curve on the final amplicons and running the amplicons on a 2% agarose gel. All qRT-PCR samples were also run on samples without reverse transcriptase to confirm the product was from mRNA, not DNA, and all of these samples showed no amplicon. Three different dilutions of cDNA were used to confirm that the samples were in the linear range.

### Western blot analysis

Cells were harvested with trypsin and then lysed using RIPA buffer (0.15 M NaCl, 50 mM Tris, pH 7.2, 1% deoxycholate, 1% Triton X-100, 0.1% SDS) containing protease inhibitors (30 μL/mL aprotinin, 4 μg/mL leupeptin, 4 μg/mL soybean trypsin inhibitor, 0.1 mM PMSF and 1 μM benzamidine). After incubation on ice for 30 min, cells were centrifuged for 10 min at 8200 × *g *to remove cellular debris. Protein concentrations were determined using the Bradford method (Bio-Rad Laboratories, Hercules, CA). Cellular protein (50 μg) was analyzed using SDS-PAGE (10–15%) and transferred to polyvinyl difluoride (PVDF) membranes. Primary antibodies were diluted 1:200 in 5% dried milk in TTBS and incubated for 1 h at room temperature. Secondary antibodies were diluted 1:5000 and incubated for 1 h at room temperature. Blots were visualized using ECL Plus reagent from GE Healthcare Life Sciences (Piscataway, NJ).

### Pathway Analysis

Data were analyzed through the use of Ingenuity Pathways (Ingenuity^® ^Systems, ). The asbestos data set with gene identifiers and corresponding expression values was uploaded into the application. Each gene identifier was mapped to its corresponding gene object in the Ingenuity Pathways Knowledge Base. A cutoff of p < 0.01 was set to identify genes whose expression was significantly differentially regulated by ± 2-fold. These genes, called focus genes, were overlaid onto a global network developed from information contained in the Ingenuity Pathways Knowledge Base. Networks of these focus genes were then algorithmically generated based on their connectivity. Biological functions associated with these focus genes were identified using the Functional Analysis using the Ingenuity Pathways Knowledge Base. Fischer's exact test was used to calculate a p-value determining the probability that each biological function assigned to the data set was due to chance alone. Networks are graphical representations of the molecular relationships between genes/gene products. Genes or gene products are represented as nodes, and the biological relationship between two nodes is represented as an edge (line). All edges are supported by at least one reference from the literature, a textbook, or from canonical pathway information stored in the Ingenuity Pathways Knowledge Base.

## Authors' contributions

LO-B carried out the cell culture and RNA preparations. BG carried out the initial analysis of the raw microarray data and aided LO-B in the clustering of the data. JRS carried out further statistical analyses in order to compare the data set to others and performed the gene ontology analyses. JPH carried out RT-PCR confirmations. AEA and JMH conceived of the study. JMH performed the Pathway Analysis, coordinated all other analyses and wrote the manuscript. All authors read and approved the final manuscript.

## Supplementary Material

Additional File 1 **Low variability in the dataset**. A comparison of the RMA expression values for the 54,675 probe sets on each of the six arrays. The tighter relationships within the Control and Treated groups are indicative of a data set with low variability. This low variability allowed the identification of a large number (2,546) of statistically significantly differentially expressed genes. Click here for file

Additional File 2 List of genes for which expression was increased or decreased two-fold or greater. Click here for file

Additional File 3 **Global test to identify specific gene ontologies related to DNA repair and damage**. A global test (Goeman et al., 2004; Goeman et al., 2005; Goeman and Oosting 2007) was used to identify specific gene ontologies related to DNA repair and damage. The p-values for each of the GO terms (abbreviated as the last four digits of each ontology's identifier) has been overlaid onto the hierarchical tree where the darkest blue node represents a p = 0.15 and the lightest represents a p = 0.016. Click here for file
