# Supplementary figures and images for: Novel functional view of the crocidolite asbestos-treated A549 human lung epithelial transcriptome reveals an intricate network of pathways with opposing functions
Source: BMC Genomics. 2008 Aug 7;9:376. doi: 10.1186/1471-2164-9-376 (PMC2533023; doi:10.1186/1471-2164-9-376)

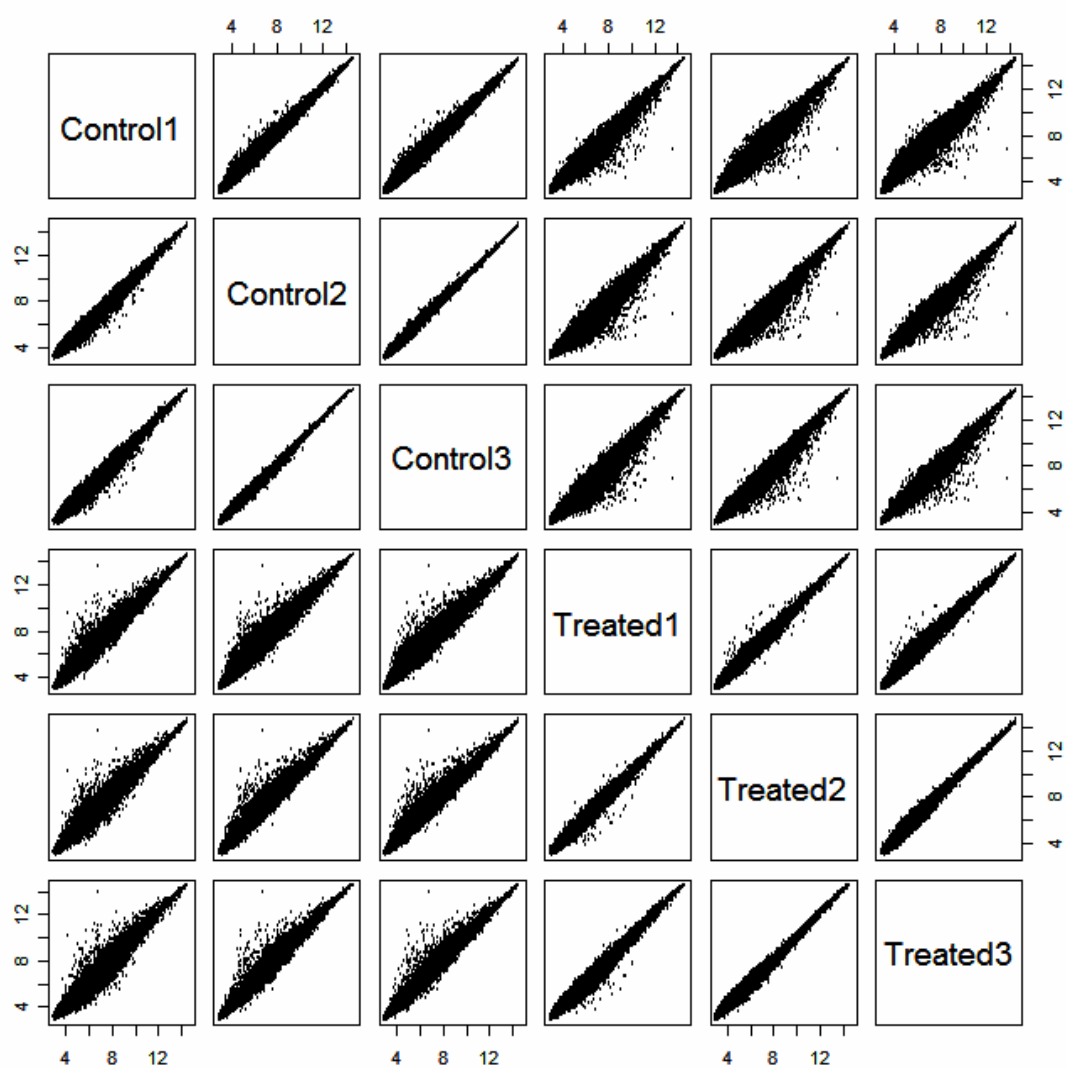

Supplement: Additional File 1 — Low variability in the dataset. A comparison of the RMA expression values for the 54,675 probe sets on each of the six arrays. The tighter relationships within the Control and Treated groups are indicative of a data set with low variability. This low variability allowed the identification of a large number (2,546) of statistically significantly differentially expressed genes. [file 1471-2164-9-376-S1.pdf]

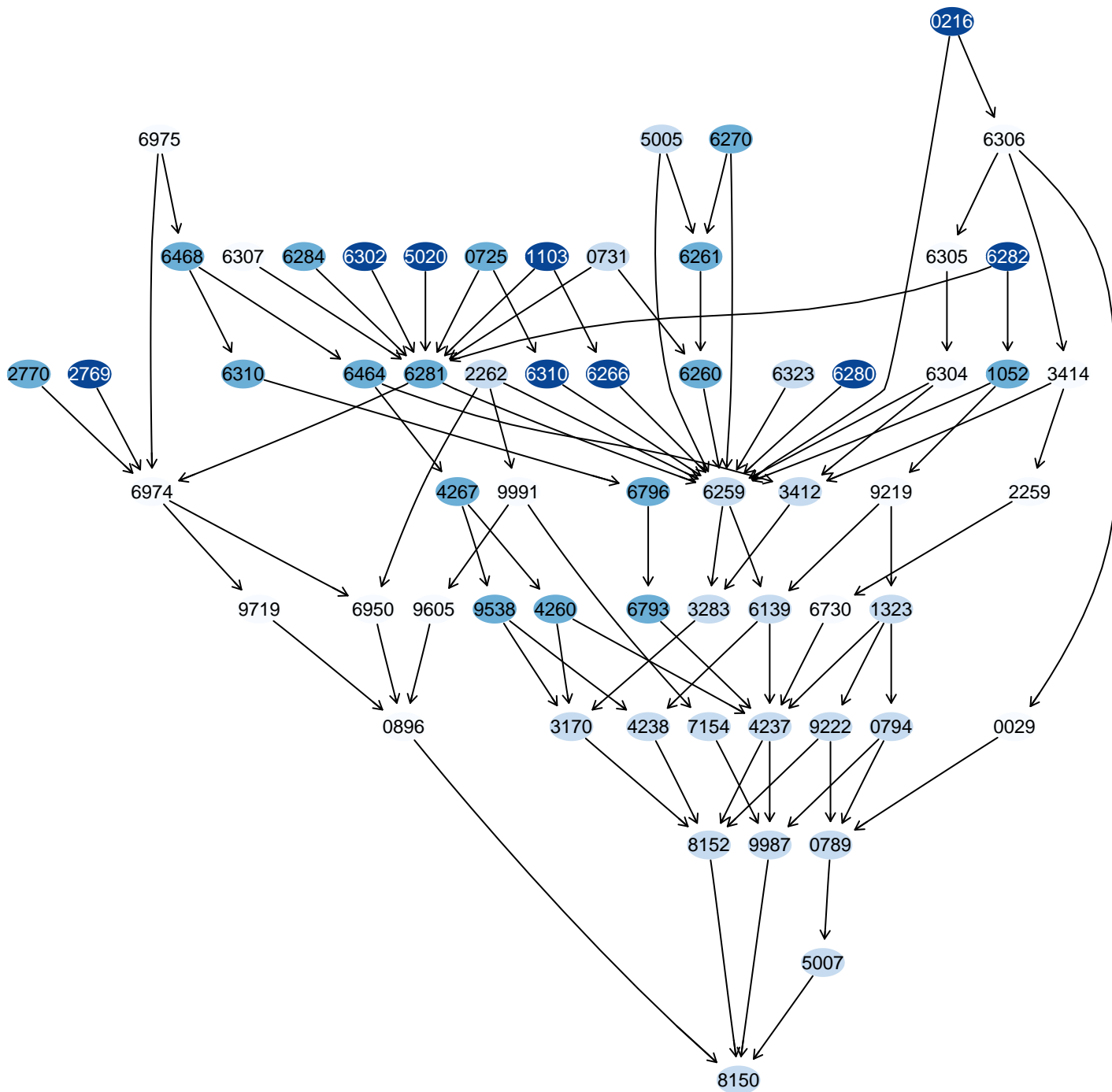

Supplement: Additional File 3 — Global test to identify specific gene ontologies related to DNA repair and damage. A global test (Goeman et al., 2004; Goeman et al., 2005; Goeman and Oosting 2007) was used to identify specific gene ontologies related to DNA repair and damage. The p-values for each of the GO terms (abbreviated as the last four digits of each ontology's identifier) has been overlaid onto the hierarchical tree where the darkest blue node represents a p = 0.15 and the lightest represents a p = 0.016. [file 1471-2164-9-376-S3.pdf]
